# Preliminary *in vivo* magnetofection data using magnetic calcium phosphate nanoparticles immobilizing DNA and iron oxide nanocrystals

**DOI:** 10.1016/j.dib.2018.04.058

**Published:** 2018-04-23

**Authors:** Quazi T.H. Shubhra, Ayako Oyane, Maki Nakamura, Sandra Puentes, Aiki Marushima, Hideo Tsurushima

**Affiliations:** a Nanomaterials Research Institute, National Institute of Advanced Industrial Science and Technology (AIST), Central 5, 1-1-1 Higashi, Tsukuba 305–8565, Japan; b Department of Intelligent Interaction Technologies, Faculty of Engineering, Information and Systems, University of Tsukuba, 1-1-1 Tennodai, Tsukuba, Ibaraki 305–8575, Japan; c Department of Neurosurgery, Faculty of Medicine, University of Tsukuba, 1-1-1 Tennodai, Tsukuba, Ibaraki 305–8575, Japan

**Keywords:** Magnetofection, Infusion fluid, Gene delivery, Calcium phosphate, Supersaturated solution

## Abstract

The data reported herein are in association with our research article entitled “Rapid one-pot fabrication of magnetic calcium phosphate nanoparticles immobilizing DNA and iron oxide nanocrystals using injection solutions for magnetofection and magnetic targeting” (Shubhra et al. 2017) [1]. This article reports morphological and gene delivery (*in vitro* and preliminary *in vivo*) data of those calcium phosphate (CaP) naonparticles (NPs) with various iron oxide (IO) contents, named as CaP-Fe(1), CaP-Fe(2), CaP-Fe(3), CaP-Fe(4), and CaP-Fe(5), which were prepared *via* coprecipitation in supersaturated CaP solutions with nominal Fe concentrations 6.97, 13.94, 27.87, 55.74, and 139.35 μg/mL, respectively. Morphological data of four different NPs: CaP-Fe(1), CaP-Fe(2), CaP-Fe(4), and CaP-Fe(5) are shown here. Data of the luciferase reporter gene expression assay show the effects of the coprecipitation time and the dosage of the CaP-Fe(3) NPs on gene expression levels of CHO-K1 cells transfected by the NPs without external magnetic field. It is demonstrated using digital and microscopic images that the CaP-Fe(3) NPs localize near the periphery of the external magnet that was placed under the cell culture plate. Using the CaP-Fe(3) NPs, animal experiments were conducted to obtain preliminary *in vivo* magnetofection data.

**Specifications table**

**Table:** 

Subject area	*Chemistry*
More specific subject area	*Materials Science, Nanotechnology, Gene Delivery*
Type of data	*Image, Graph, Figure*
How data was acquired	*Scanning electron microscope (SEM; S-4800, Hitachi-High Technologies, Inc.), optical microscope (IX71, Olympus Co.), digital camera, and luminometer (Gene Light 55, Microtec Co., Ltd.)*
Data format	*Raw and calculated data*
Experimental factors	*For SEM observation, samples were pretreated. NPs were washed, dried under vacuum and coated with carbon.*
Experimental features	*Nanoparticles (NPs) were prepared by a coprecipitation process*[1], [2], [3]*; for SEM observation, NPs were pretreated as mentioned above; for in vitro and in vivo assays, they were used after the coprecipitation process without washing.*
Data source location	*AIST, Tsukuba, 305–8565, Japan*
Data accessibility	*Data are available within this article*

**Value of the data**

• Preliminary *in vivo* magnetofection data using the calcium phosphate (CaP) nanoparticles (NPs) immobilizing DNA and iron oxide (IO) nanocrystals are presented, proving the worth of further *in vitro* and *in vivo* studies on these NPs.
• Detailed animal experimental method for *in vivo* magnetofection is described that will be helpful to design animal experiments for magnetic NPs.
• Magnetic attraction and the resulting aggregation of the NPs around the periphery of the external magnet (placed under the well) are visualized, that accounts for the unsuccessful magnetofection (magnetically enhanced gene delivery) in the standard 2D plate culture system.
• The luciferase assay data present the effects of coprecipitation time for NP preparation and dosage of the NPs on gene expression level of cells transfected by the NPs, which are useful for optimization of a similar CaP-based gene delivery system.

## 1 Data

**Fig. 1:**
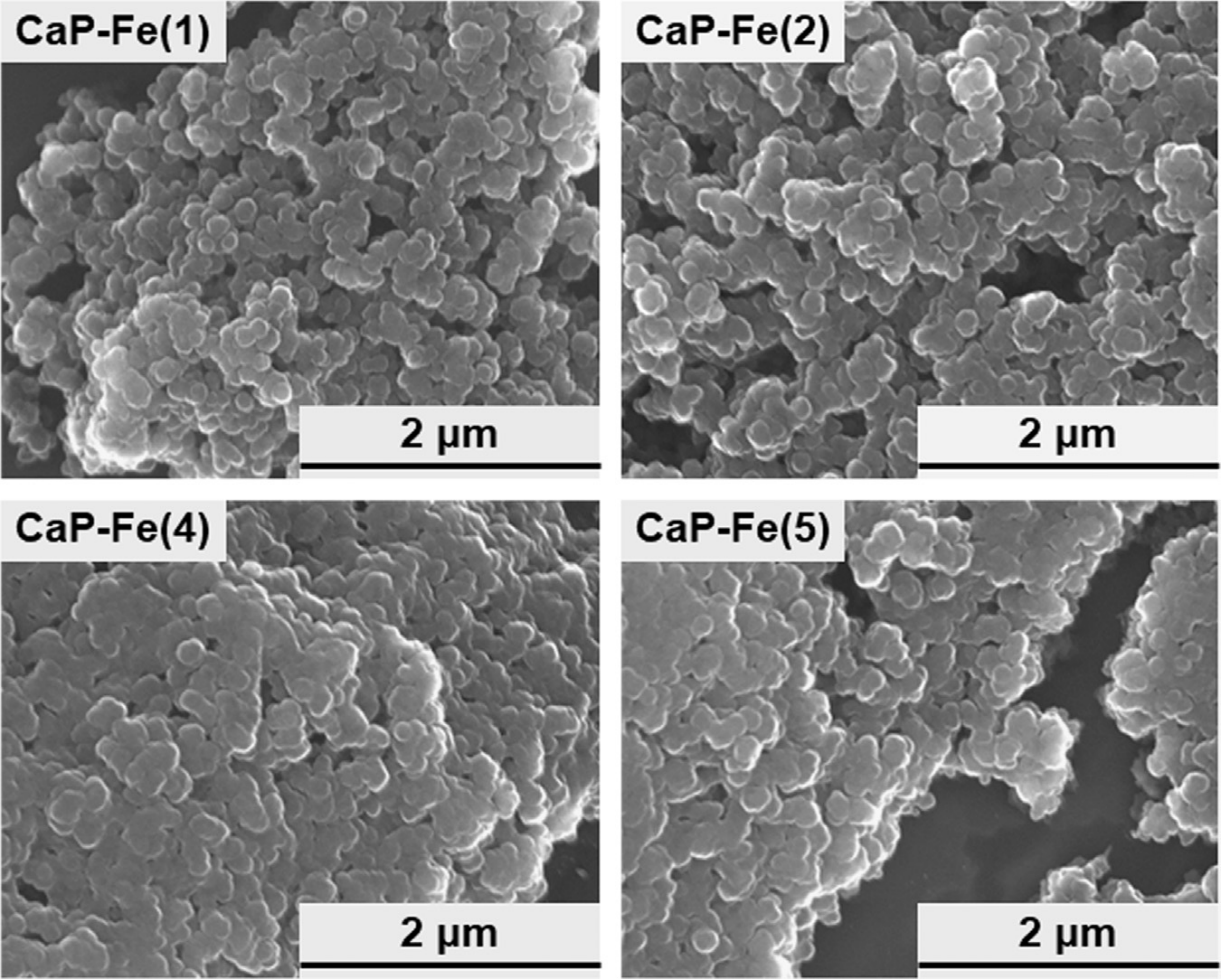
SEM images of four different NPs (CaP-Fe(1), CaP-Fe(2), CaP-Fe(4), and CaP-Fe(5)).

Fig. 1 shows scanning electron microscopy (SEM) images of four different DNA-IO-CaP NPs with various IO contents (CaP-Fe(1), CaP-Fe(2), CaP-Fe(4), and CaP-Fe(5)) [1].

**Fig. 2:**
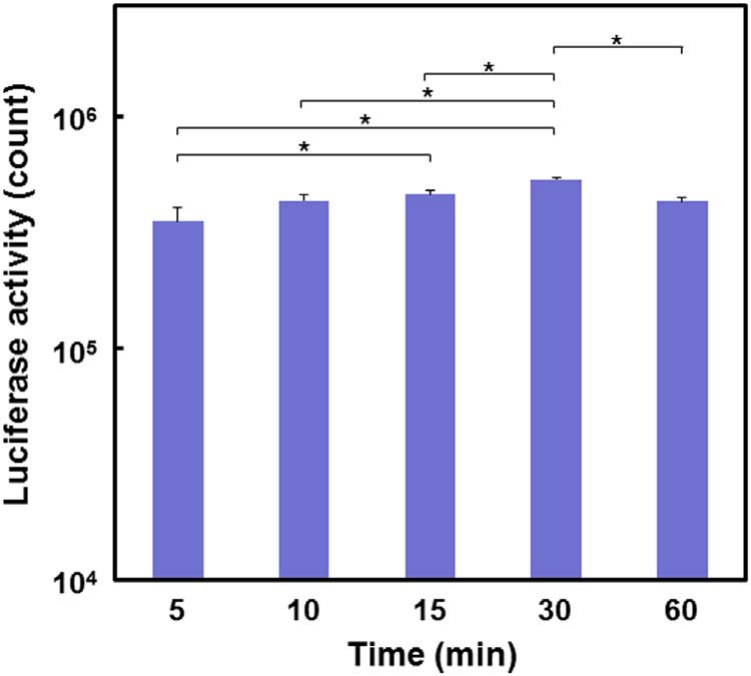
Luciferase activity of CHO-K1 cells transfected by NPs (dosage = 300 μL) that were prepared by coprecipitation for various time periods (5–60 min) in the supersaturated CaP solution that was used for CaP-Fe(3) (data presented as means + SD; *n* = 3, *p < 0.05).

**Fig. 3:**
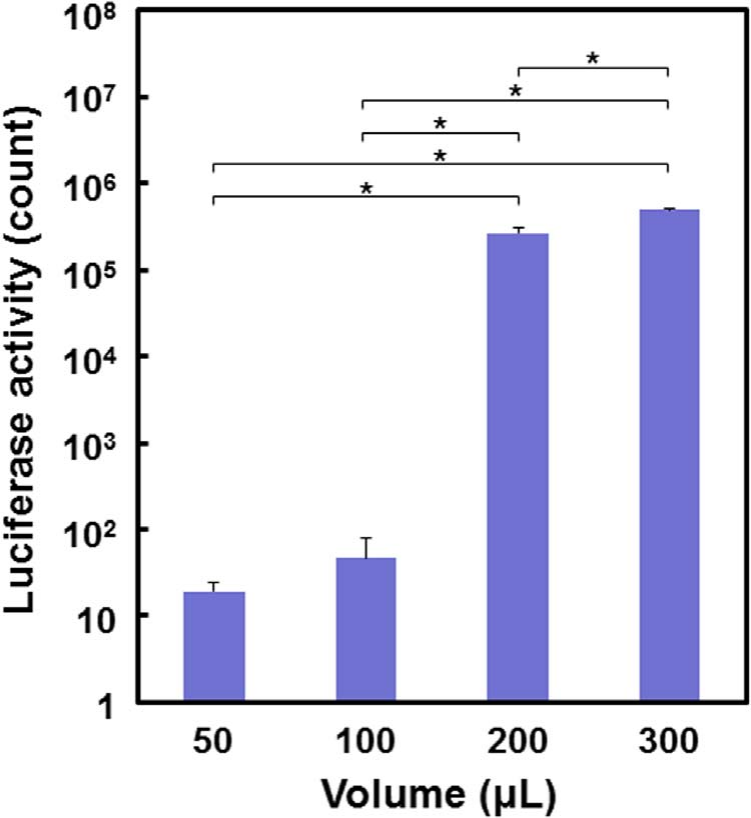
Luciferase activity of CHO-K1 cells transfected by various dosages (50–300 µL: volume of the supersaturated CaP solution with dispersed NPs) of CaP-Fe(3) (data presented as means + SD; *n* = 3, *p < 0.05).

Fig. 2 shows the effect of the coprecipitation time in the supersaturated CaP solution (the solution used for CaP-Fe(3)), whereas Fig. 3 shows that of the dosage of the CaP-Fe(3) NPs on the luciferase activity of the CHO-K1 cells transfected by NPs without external magnetic field.

**Fig. 4:**
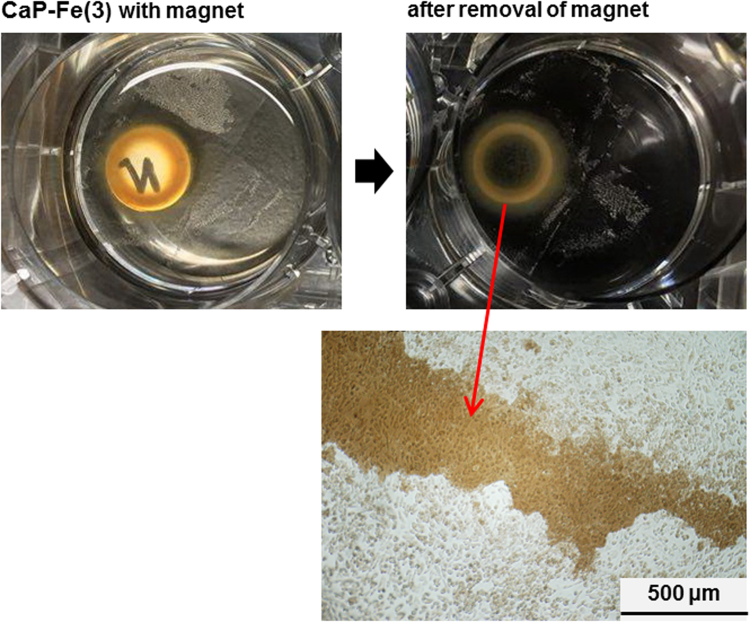
Digital (upper) and microscopic (lower) images showing aggregation of the DNA-IO-CaP (CaP-Fe(3)) NPs around the periphery of the external magnet (placed under the well in the upper left image).

Fig. 4 shows the DNA-IO-CaP (CaP-Fe(3) NPs; brown due to the immobilized IO nanocrystals) gathering and aggregating around the periphery of the external magnet that was placed under the well of a 6-well plate seeded with CHO-K1 cells.

**Fig. 5:**
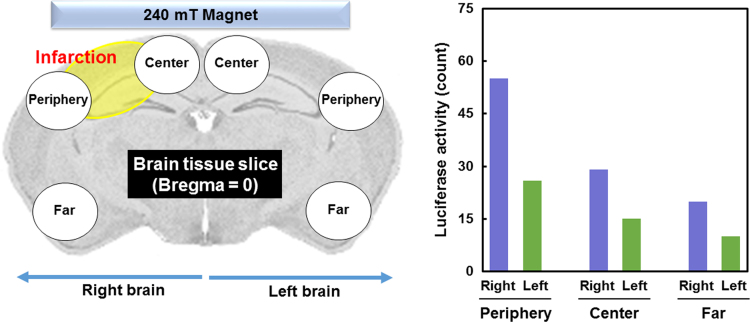
Schematic representation of the sampling position from the brain slice (left), and luciferase activity of each sample (right).

A pilot *in vivo* study was conducted to assay magnetofection and magnetic targeting capabilities of the DNA-IO-CaP (CaP-Fe(3)) NPs to the mouse ischemic brain tissue. Infarction was induced on the right side of the mouse brain, and the NPs were injected. The illustration (cross section image of the brain tissue) in Fig. 5 (left) indicates the position of the magnet and the sampling position (center of, periphery of, and far from the magnet) on the right and left sides of the brain slice (Bregma = 0). Fig. 5 (right) shows luciferase activity of each sample as a result of NP delivery to the brain tissue.

## 2 Experimental design, materials and methods

### 2.1 Preparation of NPs

DNA-IO-CaP NPs were prepared by coprecipitation in supersaturated CaP solutions and those solutions were prepared from injection solutions and plasmid including cDNA of luciferase [1].

### 2.2 SEM observation

Morphology of the prepared NPs was observed by field-emission SEM after washing, vacuum drying, and carbon coating.

### 2.3 *in vitro* transfection study without magnetic field

After coprecipitation for a certain time, the supersaturated CaP solution (50, 100, 200, or 300 μL) with dispersed NPs was added to the culture of CHO-K1 cells that were precultured for 24 h in a 24-well plate. After culturing for another 48 h, cells were assayed for luciferase activity by the protocol described in our previous papers [1], [3].

### 2.4 Pilot *in vivo* transfection study with magnetic field

Animal experiment was conducted after the approval from the animal research ethics committee of AIST and the University of Tsukuba. An eight weeks old mouse (C57BL/6 J, Charles River Laboratories Japan, Inc., Japan) was anesthetized with a mixture of ketamine (80 mg/kg) and xylazine (14 mg/kg) administered intraperitoneally. The animal's head was shaved and a 1 cm length coronal incision was done in the scalp, posterior to the eyes; following, a small spatula was used to detach the scalp from the skull posterior to the incision to create a pocket. A magnet (7 mm diameter and 1.5 mm thickness; Niroku Seisakusho Co. Ltd., Japan) of strength 240 mT was placed into the pocket completely covering the bregma and the incision was sutured. An ischemic lesion was induced by the method described by Koizumi et al. [4]. Briefly, the animal was placed over its back and the neck was shaved. A mid-line incision was made and the skin and glandular tissue were retracted to the right side. The common carotid artery (CCA) and external carotid artery were dissected and ligated permanently in their proximal end with a silk suture. A ribbon knot was placed in the distal CCA and an arteriotomy was performed in the proximal CCA, followed by the insertion of a silicone-coated monofilament (6023-PK5, Doccol Corporation, USA) which was fixed by a silk knot. The filament was inserted ~1 cm to reach the middle cerebral artery and left at place for 60 min. Then, reperfusion was achieved by removing the filament and CCA catheterization with a silicone tube was done followed by 200 μL perfusion of the supersaturated CaP solution with dispersed NPs (CaP-Fe(3)). The injection was performed over 2 min and then the catheter was removed, the proximal CCA was ligated permanently and the skin was closed with a continuous suture. The mouse was allowed to recover from anesthesia over a heating pad and housed isolated in a clean cage with access to water and soft food *ad libitum*. Forty-eight hours after injection, the mouse was sacrificed, the brain was dissected and sliced by using a brain matrix (1 mm thickness). The slice corresponding to Bregma = 0 level was used to dissect 6 samples of approximately 1 mm^3^; samples were taken bilaterally from the cortex in relation to the magnet from the center (next to the midline), periphery (lateral cortex), and far (latero-inferior) (Fig. 5 (left)). The samples were homogenized (Q125 Sonicator, Qsonica LLC., USA) at 25 °C for 30 s in a 1 mL cell lysate solution (Promega Co., USA) followed by freezing at −80 °C. Thereafter, luciferase assay was performed following the protocol described in our previous papers [1], [3].
