# Supplementary material for: Preliminary in vivo magnetofection data using magnetic calcium phosphate nanoparticles immobilizing DNA and iron oxide nanocrystals
Source: Data Brief. 2018 Apr 23;18:1696–701. doi: 10.1016/j.dib.2018.04.058 (PMC5997972; doi:10.1016/j.dib.2018.04.058)
Supplement: Supplementary file 1 — Supplementary material [file mmc1.docx]

**Confl icts of Interest Statement**

Manuscript title: **Preliminary In Vivo Magnetofection Data using Magnetic Calcium Phosphate Nanoparticles Immobilizing DNA and Iron Oxide Nanocrystals**

The authors whose names are listed immediately below certify that they have NO affiliations with or involvement in any organization or entity with any financial interest (such as honoraria; educational grants; participation in speakers’ bureaus; membership, employment, consultancies, stock ownership, or other equity interest; and expert testimony or patent-licensing arrangements), or non-financial interest (such as personal or professional relationships, affiliations, knowledge or beliefs) in the subject matter or materials discussed in this manuscript.

**Author names:** Quazi T. H. Shubhra, Ayako Oyane, Maki Nakamura, Sandra Puentes, Aiki Marushima, Hideo Tsurushima
